# Widely targeted metabolomics analysis reveals the mechanism of quality improvement of flue-cured tobacco

**DOI:** 10.3389/fpls.2022.1074029

**Published:** 2022-11-29

**Authors:** Lin Meng, Wenjing Song, Shuaiwei Chen, Fengqin Hu, Bingwen Pang, Junjie Cheng, Bing He, Fushan Sun

**Affiliations:** ^1^ Key Laboratory of Tobacco Biology and Processing, Ministry of Agriculture, Tobacco Research Institute, Chinese Academy of Agricultural Sciences (CAAS), Qingdao, China; ^2^ Technology Center, China Tobacco Shandong Industrial Co., Ltd, Jinan, China; ^3^ Excellence and Innovation Center, Jiangsu Academy of Agricultural Sciences (JAAS), Nanjing, China

**Keywords:** metabolomics, flue-curing, quality improvement, stem, tobacco

## Abstract

Flue-curing of top leaves with stems is a widely applied curing technology. Owing to the presence of stems, the quality of flue-cured leaves was significantly improved. However, the contribution of stems to flue-cured leaves is still unknown. In this study, the differences in physicochemical properties and metabolomics data between separated leaves (stem(-)) and leaves with stems (stem(+)) were investigated. The metabolic profiling of stem(+) was significantly different from that of stem(-), with phytohormone indole-3-acetic acid (IAA) being one of the most differential metabolites. The presence of stems reduced the rate of water loss in leaves, which led to less ROS accumulation, higher antioxidant enzyme activities and a lower level of membrane lipid peroxidation in stem(+) than in stem(-). The presence of stems also helped maintain the cellular membrane integrity of leaf cells by preventing the accumulation of IAA in leaf cells. Better cellular membrane integrity during flue-curing means a lower risk of leaf browning. In addition, stem(+) had a lower starch content than stem(-) because of a higher level of amylase activity. In summary, these results indicated that the presence of stems caused metabolism changes in leaves, prevented flue-cured leaves from browning and enhanced starch degradation in leaves during flue-curing.

## Introduction

Flue-cured tobacco is an important economic crop. After being harvested from the field, tobacco leaves are subjected to specific flue-curing processing, during which some complex physiological and biochemical reactions occur in flue-cured leaves. Therefore physicochemical properties of tobacco leaves and the curing method are vital factors in determining flue-cured leaf quality (Chen et al., 2019; Zong et al., 2020; Li et al., 2021). Based on the position of leaves on plants, tobacco leaves were divided into top leaves, medium leaves and bottom leaves. Compared with medium and bottom leaves, top leaves have low moisture and high dry matter content and are accessible to browning during flue-curing. Besides the flue-cured top leaves are of poor sensory quality and appearance quality. Top leaves were once considered to be of low economic value. Recently, it was found that if top leaves were flue-cured with stems, browning reaction seldom occurred during flue-curing and flue-cured leaves quality was significantly improved. In this situation, flue-cured leaves were lustrous, elastic, rich in oil and of good aroma quantity, full aroma quality, weak offensive odor and comfortable lingering smell. Flue-curing of top leaves with stems has been widely applied in production. However, the underlining mechanism of the contribution of stems to top leaves is still unknown.

The chemical composition of flue-cured tobacco leaves is very complex, in which thousands of chemical compounds have been identified (Kaiser et al., 2018). Flue-cured tobacco leaf quality is highly related to chemical compositions (Talhout et al., 2006). Therefore, it is necessary to study the influence of different curing methods on flue-cured tobacco leaf quality by quantifying important compounds related to quality. Generally, it is unrealistic to quantify and determine each compound in separate measurements. That is time and cost-consuming. Metabolomics is a technique for large-scale detection of all metabolites in a biological system, like whole organisms, tissues, or individual cells. Metabolomics study includes targeted metabolomics, untargeted metabolomics, and widely targeted metabolomics. Widely targeted metabolomic analysis is a novel approach combining non-targeted and targeted metabolomics advantages (Chen et al., 2013). It has the characteristics of high throughput, ultra sensitivity, wide coverage, and accurate qualitative and quantitative analysis. Metabolomics has been used to characterize the quality and composition of flue-cured tobacco leaves under different conditions (Zhao et al., 2014; Zhao et al., 2018; Hu et al., 2021). However, the influence of stem on metabolic profiling of flue-cured tobacco leaves has not yet been reported.

The flue-curing process is divided into three stages: yellowing stage, leaf drying stage and stem drying stage. In the yellowing stage, macromolecular substances are decomposed into small molecule substances to form aroma precursors. The yellowing stage is crucial for improving flue-curd leaves characteristics and the critical stage of flue-cured leaves appearance quality formation. During the yellowing stage, separated top leaves changed easily from yellow to brown, severely reducing the flue-cured leaves’ quality. In this study, we investigated the contribution of stems to the quality of flue-cured top leaves by analyzing the differences in physicochemical properties and metabolomics data between separated leaves and leaves with stems. Leaf samples were collected at the end of the yellowing stage. Metabolomics analysis was performed with the widely targeted method. Based on the MWDB database, more than 3000 metabolites could be determined in a measurement. Our results will provide a theoretical basis for further optimizing the flue-curing technology of top leaves.

## Materials and methods

### Materials

**Figure 1:**
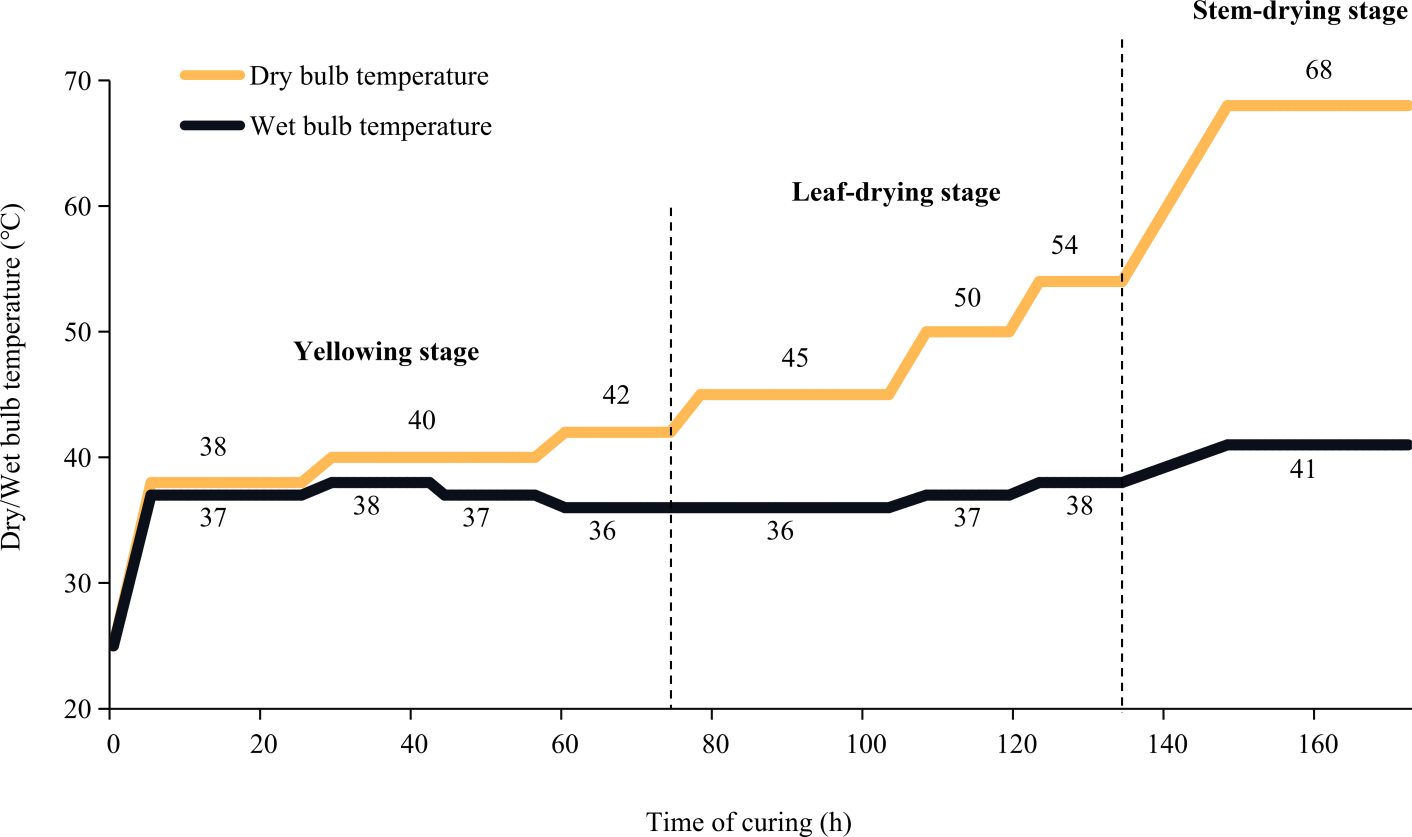
Flue-curing method.

Tobacco genotype “Yunyan87” was seeded in seedling trays in a greenhouse. The 50-day-old seedlings were then transplanted into fields located in Yunnan, China (98°68′E, 24°56′N) on 20 April 2021. Pure nitrogen at 105 kg/ha was applied in the field period and fertilizer at N: P_2_O_5_: K_2_O (1:1:2.5) was applied. 50% of the total amount was used as base fertiliser, 15% was used as seedling promoting fertilizer, and the other 35% was additional fertilizer. At the time of top pruning (9 July 2021), tobacco plants’ apical buds were removed, and 15 to 16 leaves per plant were left. At the maturing stage (5 September 2021), the top five leaves with stems were harvested. Two groups of flue-cured samples (leaf with stem, stem(+), and leaf without stem, stem(-)) were prepared. Stem(+): Leaves with stems were flue-curd. After curing, stems were separated from leaves, with leaves being used for further metabonomics and physicochemical analysis. Stem(-): Stems were separated from leaves before flue-curing. Only leaves were flue-cured and used for further metabonomics and physicochemical analysis. Additionally, the fresh leaves (stems were removed) were also used as the control sample (CK). Three biological replicates were generated for each group of samples (9 samples in total). The flue-curing procedure is shown in **Figure 1**.

The samples of tobacco leaves were collected at the end of the yellowing stage. After removing the main vein, leaves were put in liquid nitrogen immediately and then transferred to the -80°C fridge for further analysis.

### Physicochemical analysis

#### Moisture content of tobacco leaves

To determine moisture content of leaves, 10 leaves from each group were weighed immediately after harvest (fresh weight, FW). Then the leaves were dried in the oven at 70°C for 24 h to obtain dry weight (DW). Moisture content was calculated using the following formula.


Moisture content=(FW−DW)/FW×100

#### H_2_O_2_, O_2_-, MDA and starch contents

H_2_O_2_, O_2_-, MDA and starch contents were determined with commercially available assay kit (Suzhou Comin Biotechnology Co., Ltd., China).

#### Enzyme activities

Activities of superoxide dismutases (SOD), catalase (CAT), glutathione reductase (GR), polyphenol oxidase (PPO) and total amylase were determined with commercially available assay kit (Suzhou Comin Biotechnology Co., Ltd., China).

#### Widely targeted metabolomics analysis

Leaf samples (stem(+), stem(-) and CK) were vacuum freeze-dried and milled. Then, 100 mg lyophilized powder was extracted with 1.2 mL 70% methanol, vortexed for 30 s every 30 min for 6 times in total, and stored at 4°C overnight. Following centrifugation at 10,000 rpm for 10 min, the supernatants were filtrated through a 0.22 μm membrane. Finally, sample extracts were analyzed using a UPLC-ESI-MS/MS system (UPLC, SHIMADZU Nexera X2; MS, Applied Biosystems 6500 Q TRAP).

Column (1.8 μm, 2.1 mm × 100 mm, Agilent SB-C18); solvent system, water (0.1% formic acid) and acetonitrile (0.1% formic acid); gradient program, 95:5 v/v at 0 min, 5:95 V/V at 9 min, hold for 1 min, 95:5 v/v at 11.1 min, hold for 2.9 min; flow rate, 0.35 mL/min; column temperature, 40°C; injection volume, 2 μL. The effluent was alternatively connected to an ESI-triple quadrupole-linear ion trap (Q TRAP)-MS.

Linear ion trap (LIT) and triple quadrupole (QQQ) scans were acquired form the Applied Biosystems 6500 Q TRAP UPLC/MS/MS System, which was equipped with an ESI Turbo Ion-Spray interface. Positive and negative ion modes are operated by Analyst 1.6.3 software (AB Sciex). The ESI source operation parameters were as follows: ion source, turbo spray; source temperature, 550°C; ion spray voltage (IS), 5500 V (positive ion mode)/-4500 V (negative ion mode); ion source gas I (GSI), gas II (GSII), curtain gas (CUR) were set at 50, 60, and 25.0 psi, respectively; the collision-activated dissociation (CAD) was set to high. Instrument tuning and mass calibration were performed with 10 and 100 μM polypropylene glycol solutions in QQQ and LIT modes, respectively. QQQ scans were performed through multiple reaction monitoring (MRM) model, with collision gas (nitrogen) set to medium. Declustering potential (DP) and collision energy (CE) for each MRM transition were determined through optimization. A specific set of MRM transitions were monitored for each period, according to the metabolites eluted within this period.

#### Qualitative and quantitative analysis

Metabolites were identified based on the Metware database (MWDB) created by MetWare Biotechnology Co., Ltd. (Wuhan, China) using secondary mass-spectrometry data. The interference from isotope signals, duplicate signals of K^+^, Na^+^, and $NH_{4}^{+}$ ions, as well as duplicate signals of fragment ions derived from other larger molecules, were excluded.

Metabolites were quantified using the MRM model of QQQ mass spectrometry. In MRM mode, the quadrupole firstly searched for precursor ions (parent ions) of target substances to eliminate the interference from the ions derived from substances of different molecular weights. The precursor ions were then fragmented *via* induced ionization in the collision chamber into many fragment ions, from which a characteristic ion was selected. Using characteristic ions eliminated the interference from non-target ions and made more precise and repeatable quantification results. When metabolite mass spectrometry data were obtained for different samples, mass spectrum peaks of all metabolites were subjected to area integration. The mass spectrum peaks of the same metabolite in different samples were subjected to integration correction. The relative quantity of a metabolite was represented by the area of its corresponding mass spectrum peak.

#### Multivariate statistical analysis

Principal component analysis (PCA) analysis was performed using the Clustvis tool (Metsalu and Vilo, 2015). Orthogonal partial least square-discriminant analysis (OPLS-DA) was generated using the R package MetaboAnalystR. To avoid overfitting, a permutation test (200 permutations) was performed. Boxplot, heatmap and clustered dendrogram were drawn using the R package.

#### Differential metabolite analysis

Two screening criteria for differential metabolites were established: p-value< 0.05; variable importance in projection (VIP) was ≥ 1. VIP values were extracted from the OPLS-DA result.

### Results and discussion

#### Effect of stem on tobacco leaves metabolome

**Figure 2:**
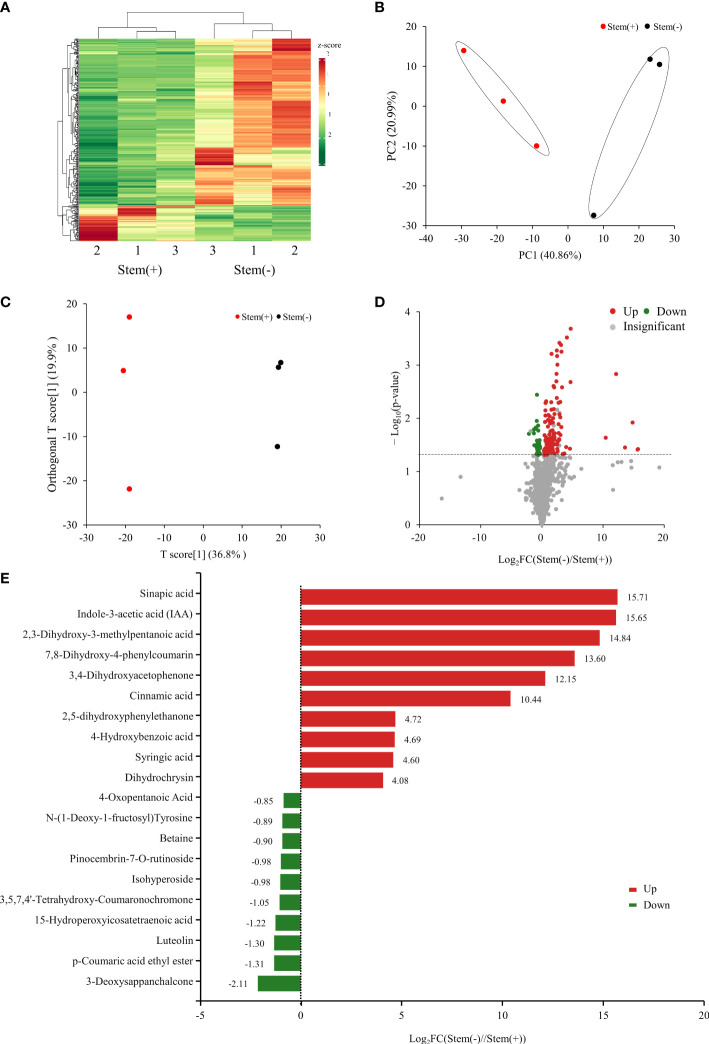
Metabolome analysis of tobacco leaves. **(A)** Heatmap of relative metabolites concentrations. The clustered dendrogram of samples was on the top. **(B)** PCA score plot of metabolite profiles. **(C)** Score scatter plots of the OPLS-DA model for stem(+) versus stem(-). **(D)** Volcano plot of differential metabolites between stem(+) and stem(-). **(E)** Top 20 differential metabolites between stem(+) and stem(-). FC, fold change.

Metabolome analysis of flue-cured tobacco leaves was carried out based on widely targeted metabolomics analysis using the self-built database MWDB. Totally, 1236 metabolites were identified in flue-cured tobacco leaves, with 1213 and 1234 metabolites being identified in stem(+) and stem(-), respectively. The quantities of metabolites were subjected to hierarchical clustering (**Figure 2A**) and PCA analysis (**Figure 2B**). They both showed two distinct groups corresponding to stem(+) and stem(-) samples, respectively. Stem(+) and stem(-) were clearly separated by the first component (PC1: 40.86% variance explained). There was obviously distinct grouping of the samples based on whether to reserve stems. To evaluate the metabolic alteration caused by the presence of stems, OPLS-DA was performed. As shown in the OPLS-DA score plot, stem(+) and stem(-) were well separated. The model parameters were R^2^X = 0.567, R^2^Y = 0.999, and Q^2^ = 0.8 (**Figure 2C**). 160 metabolites significantly differed (VIP ≥ 1, p< 0.05) between stem(+) and stem(-). Compared with stem(+), 126 metabolites were up-regulated and 34 were down-regulated (**Figure 2D**). Sinapic acid was the most up-regulated metabolite between stem(+) and stem(-) (**Figure 2E**). Indole-3-acetic acid (IAA) was the second most differential metabolites, which was abundant in stem(-) but did not exist in stem(+). 3-deoxysappanchalcone was the most down-regulated metabolite, followed by p-coumaric acid ethyl ester.

#### Effect of stem on the moisture content of tobacco leaves

**Figure 3:**
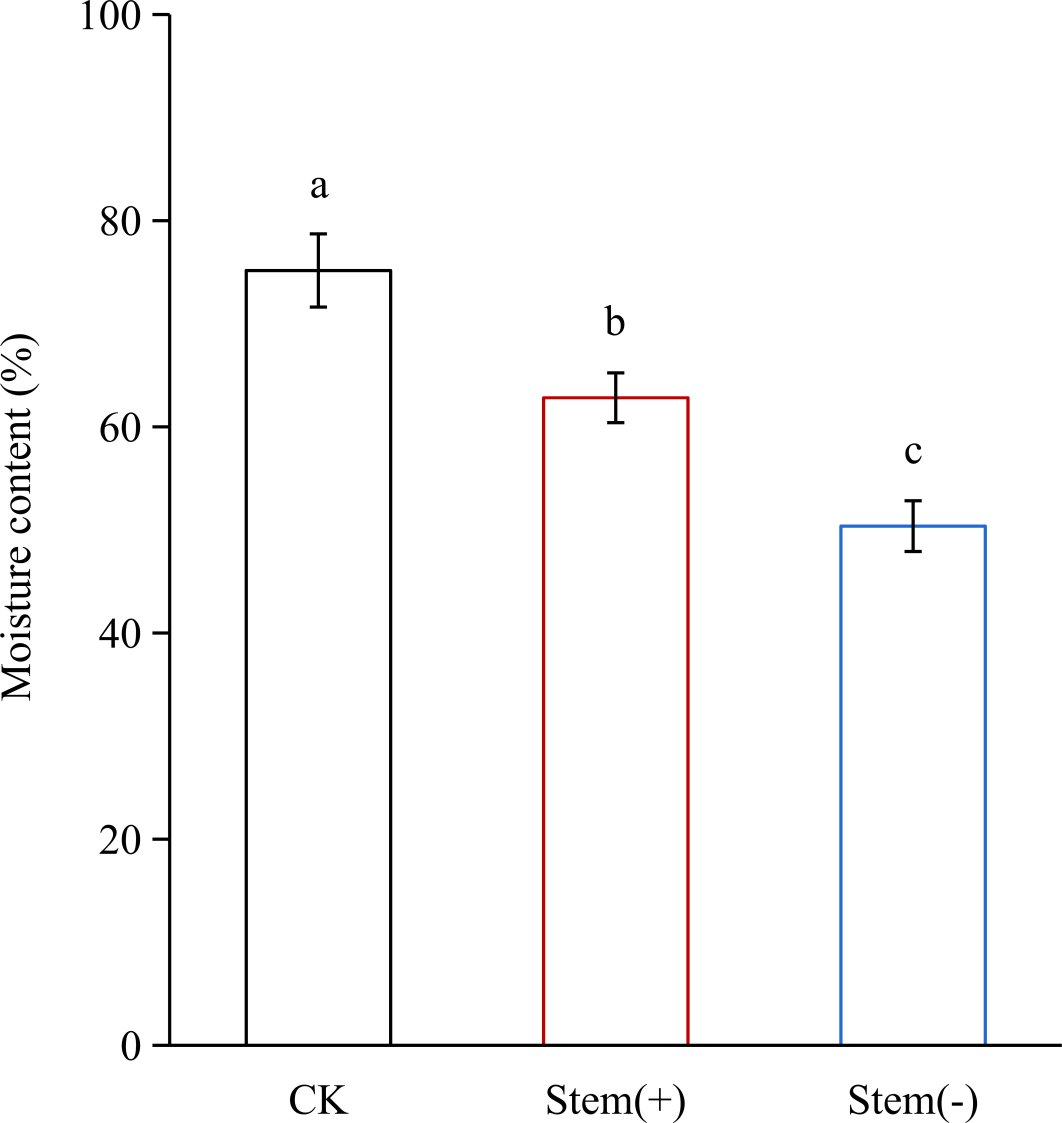
Effect of stem on leaf moisture content in flue-cured tobacco leaves. Different letters indicate statistically significant difference at p < 0.05, as determined by t-test.

During flue-curing, tobacco leaves were dehydrated. At the end of the yellowing stage, the leaf moisture content decreased significantly from 75.17% in CK to 62.82% and 50.37% in stem(+) and stem(-), respectively (**Figure 3**). This result agreed with a previous study that the presence of stem could reduce leaves’ water loss during the withing process (Zeng et al., 2017; Wei et al., 2018). In our study, stem(+) and stem(-) were processed by the same curing method with the only difference being whether leaves were processed with stems. The moisture content decreased more slowly (p< 0.05) in stem(+) than in stem(-), because water migrated from the stem to the leaf *via* the midrib during flue-curing (Wei et al., 2018), which reduced the dehydration rate of tobacco leaves.

#### Effect of stem on H_2_O_2_ content, O_2_- content in tobacco leaves

**Figure 4:**
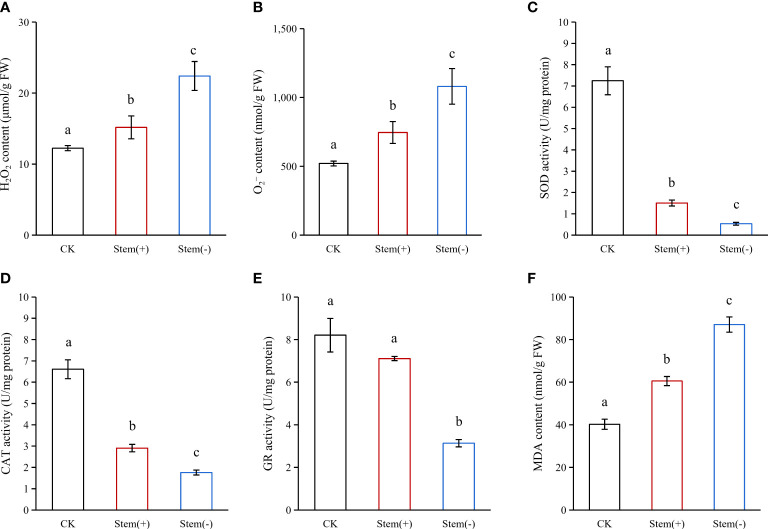
Effect of stem on ROS, antioxidant enzymes and cell integrity in flue-cured tobacco leaves. FW, fresh weight. Different letters indicate statistically significant difference at p < 0.05, as determined by t-test.

During flue-curing, tobacco leaves were subjected to various stresses such as heat and drought stresses. Such stresses could lead to reactive oxygen species (ROS) accumulation at the cellular level (Bouchard and Purdie, 2011; Petrov et al., 2015; Medina et al., 2021). H_2_O_2_ and $O_{2}^{-}$ are essential components of ROS (Gill and Tuteja, 2010). Both of them significantly accumulated in flue-cured leaves (**Figure 4**). Both H_2_O_2_ and $O_{2}^{-}$ contents were significantly higher in stem(-) than in stem(+). The H_2_O_2_ content in stem(-) was 48% higher than that in stem(+) (**Figure 4A**), while the $O_{2}^{-}$ content was 45% higher in stem(-) than in stem(+) (**Figure 4B**). The difference in ROS contents between stem(+) and stem(-) was attributed to the difference in leaf moisture content. Our results showed a decrease in tobacco leaf moisture content was accompanied by increases in H_2_O_2_ and $O_{2}^{-}$ concentrations. Such association between ROS contents and leaf water deficit has been observed in other species, such as maize and wheat (Khanna-Chopra and Selote, 2007; Yao et al., 2013). The presence of stem attenuated ROS accumulation in flue-cured tobacco leaves.

#### Effect of stem on SOD, CAT and GR activity in tobacco leaves

The overproduction of ROS can cause oxidative cell damage, directly attack membrane lipids, inactivate metabolic enzymes and damage the nucleic acids (Mittler, 2002). Plants have developed antioxidant defense systems to cope with the ROS generation to maintain intracellular homeostasis, consisting of antioxidant enzymes like SOD, CAT and GR and non-enzymatic antioxidants like GSH.

SOD is the most effective intracellular enzymatic antioxidant, which catalyzes the dismutation of $O_{2}^{-}$ to H_2_O and O_2_ (Gill and Tuteja, 2010). The SOD activity could increase under mild water deficiency and decrease under severe water deficiency (Jiang and Huang, 2001; Yao et al., 2013). The activity of SOD in stem(+) and stem(-) was 81% and 93% below that in CK (**Figure 4C**), showing that the combined stresses (heat stress and drought stress) during the flue-curing process greatly limited SOD activity and impaired $O_{2}^{-}$ scavenging in the cell (Jiang and Huang, 2001). The SOD activity in stem(+) was considerably higher than in stem(-), indicating that SOD activity is proportional to leaf tissue moisture content under severe water deficit conditions in tobacco leaves.

CAT dismutases H_2_O_2_ into H_2_O and O_2_ and are indispensable for ROS scavenging during stress conditions (Garg and Manchanda, 2009). CAT activity could be up-regulated, unaffected or down-regulated by water deficit (Jiang and Huang, 2001; Pan et al., 2006; Yao et al., 2013). Like SOD, CAT activity declined significantly in flue-cured tobacco leaves (**Figure 4D**). Compared to CK, the activity of CAT decreased by 56% and 73% in stem(+) and stem(-), respectively, which favored the accumulation of H_2_O_2_. The CAT activity in stem(-) was about 60% as much as that in stem(+), showing that the decline of CAT activity increases with the degree of water deficit in flue-cured tobacco leaves.

GR converts oxidized glutathione (GSSG) to reduced GSH, thus helping maintain the GSH pool and reducing the environment in the cell, which is crucial for the active functioning of proteins (Trivedi et al., 2013). The response of GR to stresses varied due to species variation. In *Sedum album* L., GR activity increased even under severe water deficit, whereas in turfgrasses, GR activity decreased under severe water deficit (Castillo, 1996; Jiang and Huang, 2001). Flue-curing tended to reduce the activity of GR in stem(+), but this effect was not statistically significant (**Figure 4E**). However, flue-curing significantly reduced the activity of GR in stem(-). Our results showed that flue-curing inhibited the GR activity in tobacco leaves, and the effect increased with decreasing leaf moisture content.

Due to flue-curing, the activities of SOD, CAT and GR decreased, which contributed to the accumulation of ROS in tobacco cells. However, the ROS scavenging system was relatively less damaged in stem(+), because of the higher leaf moisture content. The presence of stems alleviated ROS scavenging system damage in leaves, which probably led to less ROS accumulation in stem(+).

#### Effect of stem on MDA content in tobacco leaves

Accumulation of ROS can induce membrane lipid peroxidation. MDA is the end product of membrane lipid peroxidation and the indicator of biomembrane integrity (Wongsheree et al., 2009). The higher the MDA content, the more severe the biomembrane is injured. The MDA content increased by 50% and 116% in stem(+) and stem(-), respectively, compared to CK. The MDA content was enormously higher in stem(-) than in Stem(+) (**Figure 4F**). This is because the dehydration rate was more rapid in stem(-), leading to lower antioxidant enzyme activities and higher ROS accumulation (Jiang and Huang, 2001; Su et al., 2017; Zhao et al., 2022). Our observation showed that separated leaves (stem(-)) underwent a higher level of membrane lipid peroxidation than leaves with stems (stem(+)) during flue-curing.

#### Effect of stem on polyphenol contents in tobacco leaves

Polyphenols are secondary metabolites produced by plants such as tobacco. They play an essential role in determining the flue-cured tobacco quality, principally the color and aroma of flue-cured leaves and the odor and taste of smoke (Gong et al., 2006; Dagnon et al., 2006). During flue-curing of separated top tobacco leaves, however, polyphenols were easily oxidized into quinone by PPO to form brown-colored substances, which cause cured leaf browning and reduce flue-cured tobacco quality (Chen et al., 2021; Zhao et al., 2022). In tobacco, polyphenols exist in the form of glucoside and ester (Romero et al., 2004). The polyphenol contents dramatically change during flue-curing, because of the pyrolysis and the enzymatic degradation of the phenolic glycoside. Chlorogenic acid, rutin, and scopoletin are major polyphenols in tobacco leaves. Metabolome data showed that the chlorogenic acid content and rutin content did not differ significantly among CK, stem(+) and stem(-), whereas the scopoletin content showed an increasing trend due to flue-curing (**Table 1**). The scopoletin content was significantly higher in stem(-) than in stem(+), suggesting that its accumulation was probably related to the dehydration rate in tobacco leaves.

**Table 1:** Polyphenols contents in flue-cured tobacco leaves.

Sample	Chlorogenic acid	Scopoletin	Rutin
CK	7.03E+06 ± 1.11E+06 a	1.03E+07 ± 2.60E+06 b	6.48E+07 ± 1.62E+07 a
Stem(+)	8.82E+06 ± 1.24E+06 a	1.59E+07 ± 3.68E+06 b	7.60E+07 ± 1.23E+07 a
Stem(-)	8.11E+06 ± 2.56E+05 a	6.42E+07 ± 7.64E+06 a	5.98E+07 ± 1.72E+07 a

The relative quantity of a metabolite was represented by the area of its corresponding mass spectrum peak.

#### Effect of stem on PPO activity in tobacco leaves

**Figure 5:**
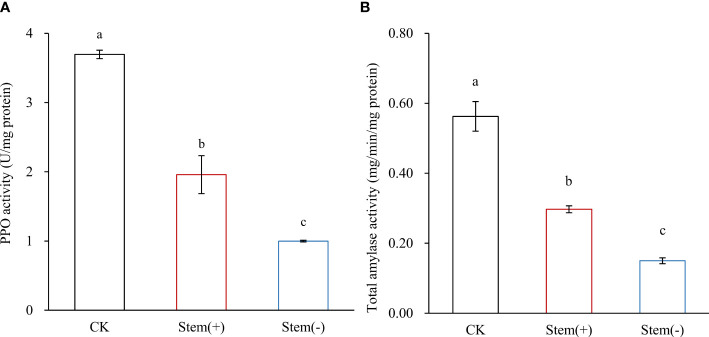
Effect of stem on PPO activity and total amylase activity in flue-cured tobacco leaves. Different letters indicate statistically significant difference at p < 0.05, as determined by t-test.

PPO catalyzes the oxidation of polyphenols to quinones. In tobacco, PPO mainly exists in chloroplasts (Lax and Vaughn, 1991; Mayer, 2006). Previous studies showed that PPO activity positively correlates with the environmental moisture content during tobacco leaf processing (Song et al., 2010; Zhao et al., 2022). In our study, the moisture content of tobacco leaves decreased in the following order: CK > stem(+) > stem(-). The PPO activity was the lowest in the stem(-), about 30% and 50% that of CK and stem(+), respectively, and the differences among the three groups of samples were statistically significant (**Figure 5A**). That is, PPO activity in stem(-) was lower than that in stem(+), because of the lower moisture content in stem(-).

At the end of the yellowing stage, tobacco leaf moisture content is high. Thus, cells in leaves still had high PPO activity. Typically, tobacco leaves undergo a low degree of polyphenols oxidation at this moment, because the intracellular membrane system provides a barrier between the PPO and polyphenols. In stem(-), rapid dehydration sharply increased membrane lipid peroxidation levels and consequently reduced the integrity of the cell membrane. If the membrane system destructed due to a high level of membrane lipid peroxidation, polyphenols would come into contact with PPO and much oxygen would enter cells, which increases the oxidation rate of polyphenols to form brown substances (Chen et al., 2019; Zhao et al., 2022). Such browning reactions were to cause the appearance of dark-colored spots and stripes on the leaf surface and seriously reduce flue-cured tobacco leaf quality. Unlike stem(-), stem(+) had a low risk of browning reaction because of a suitable dehydration rate and relatively intact cellular membrane system. Due to the unique physicochemical properties of tobacco top leaves, the browning reaction can quickly occur during flue-curing of separated top leaves, making them have low economic value. Nevertheless, browning reaction seldom occurred during flue-curing of top leaves with stems. Our observation suggested that the presence of stem helped maintain suitable leaf moisture content and cell membrane integrity during the yellowing stage, reducing the risk of browning reaction.

#### Effect of stem on phytohormone contents of tobacco leaves

Based on our metabolome data, four plant hormones, salicylic acid (SA), indole-3-acetic acid (IAA), jasmonic acid (JA) and abscisic acid (ABA), were identified in tobacco leaves (**Table 2**). IAA and SA were significantly different between stem(+) and stem(-), probably due to the different moisture content.

**Table 2:** Phytohormone contents in flue-cured tobacco leaves.

Sample	IAA	JA	SA	ABA
CK	9 ± 0 b	7.39E+03 ± 1.28E+04 c	3.66E+05 ± 2.19E+05 c	1.31E+05 ± 2.44E+04 b
Stem(+)	9 ± 0 b	8.32E+03 ± 1.44E+04 bc	6.23E+05 ± 1.91E+05 bc	4.76E+05 ± 2.57E+04 a
Stem(-)	4.61E+05 ± 1.62E+05 a	5.51E+04 ± 9.91E+03 a	1.28E+06 ± 4.49E+05 ab	5.98E+05 ± 8.56E+04 a

The relative quantity of a metabolite was represented by the area of its corresponding mass spectrum peak.

IAA was one of the most differential metabolites between stem(+) and stem(-). Stem(+), as well as CK, did not contain IAA, but stem(-) accumulated a high level of IAA, which is probably due to the leaf moisture content of stem(-) being significantly lower than that in stem(+) and CK. It is reasonable to speculate that IAA was synthesized in separated tobacco leaves during flue-curing. This is the first time the synthesis and accumulation of auxin are observed during tobacco leaf flue-curing. IAA is the main naturally occurring auxin, which controls plant growth and development *via* promoting cell division (proliferation), growth (expansion, elongation) and differentiation (Mor et al., 2002). Various studies have shown that during cell expansion and elongation, IAA causes cell wall loss by promoting xyloglucan degradation (Mor et al., 2002; dos Santos et al., 2004; Mateusz and Robert, 2018). The degradation of cell wall substances made plastids and membrane systems vulnerable. The cell membrane integrity was poor in stem(-) due to a high level of membrane lipid peroxidation. IAA accumulation further increased the risk of cell membrane destruction. At the end of the yellowing stage, PPO activity in leaves was still high. If the cell membrane were disrupted, polyphenols would be oxidized by PPO, causing a browning reaction (Chen et al., 2019; Zhao et al., 2022). Therefore the accumulation of IAA was probably another reason for the frequent occurrence of browning reaction during flue-curing of separated top leaves. The presence of stem probably inhibited or postponed IAA accumulation in flue-cured leaves, which awaits further study.

#### Effect of stem on carbohydrates contents of tobacco leaves

The starch content is an important indicator of the quality of flue-cured tobacco leaves. Starch adversely affects the combustion rate and complete combustibility of cigarettes and produces a bitter and irritating taste when it burns. Starch degradation in tobacco leaves during the curing process is predominantly catalyzed by amylolytic enzymes, which are sensitive to leaf moisture content (Song et al., 2009; Yamaguchi et al., 2013). Low humidity can significantly reduce their activity in tobacco leaves (Song et al., 2009). Therefore, it is essential to maintain the appropriate leaf moisture content during flue-curing processing to degrade starch fully. Compared to CK, both starch content and total amylase activity significantly decreased in flue-cured leaves (**Figure 5B**, **Table 3**). The starch content was significantly higher in stem(-) than in stem(+), while the total amylase activity was significantly lower in stem(-) than in stem(+). These results coincided with the prediction, because leaf moisture content was lower in stem(-) than in stem(+). Compared to stem(+), the moisture content decreased rapidly in stem(-), resulting in lower total amylase activity and less starch degradation. In conclusion, stems could slow the decrease of amylase activity and strengthen starch degradation by maintaining appropriate leaf moisture content, which is advantageous to flue-cured tobacco qualities.

**Table 3:** Carbohydrates contents in flue-cured tobacco leaves.

Sample	Starch(mg/g FW)	Glucose*	Fructose*	Sucrose*	Maltose*
CK	212.34 ± 15.79 a	7.41E+06 ± 8.86E+05 b	9.54E+06 ± 1.48E+06 b	3.12E+07 ± 9.29E+06 b	2.90E+06 ± 7.92E+05 a
Stem(+)	112.58 ± 5.21 c	1.86E+07 ± 1.52E+06 a	2.20E+07 ± 4.95E+06 a	5.66E+07 ± 5.12E+06 a	4.66E+06 ± 1.26E+06 a
Stem(-)	158.24 ± 11.81 b	2.08E+07 ± 1.34E+06 a	2.19E+07 ± 6.67E+06 a	7.26E+07 ± 2.14E+07 a	6.58E+06 ± 2.20E+06 a

FW, fried weight. *, The relative quantity of a metabolite was represented by the area of its corresponding mass spectrum peak.

Water-soluble sugars also contribute to the quality of cured tobacco leaves. Cured tobacco leaves with high water-soluble sugars are lustrous, elastic, rich in oil, elegant flavor, and mellow taste. It is usually considered that the main ingredient of soluble sugar in flue-cured leaves are glucose, fructose, sucrose and maltose (Banožić et al., 2020). Our metabolome analysis showed that glucose, fructose and sucrose contents significantly increased during processing, while maltose content remained almost the same (**Table 3**). However, there was no significant difference in glucose, fructose, sucrose and maltose contents between stem(+) and stem(-). These results indicated that the presence of stem did not significantly improve the water-soluble sugar content of leaves.

## Conclusion

To reveal the underlining mechanism of the contribution of stems to leaves, this paper investigated the effects of stem on the metabolic profiling and physicochemical properties of flue-cured tobacco leaf at the end yellowing stage. Stems reduced the dehydration rate of leaves, which led to less ROS accumulation, higher antioxidant enzyme activities and a lower level of membrane lipid peroxidation in stem(+). Stem also prohibited IAA accumulation in leaves, which probably be related to the difference in leaf moisture content between stem(+) and stem(-). IAA accumulation could make the cellular membrane system vulnerable to external damage. That is stems helped maintain cellular membrane integrity of leaf cells through alleviating oxidative membrane damage and inhibiting IAA accumulation, which reduced the risks of browning reaction in flue-cured leaves. Stems enhanced starch degradation in leaves, which was favorable to sensory quality of flue-cured leaves. The activity of amylase in stem(+) was higher than that in stem(-), because leaf moisture content was higher in stem(+). Stems caused various metabolic and physicochemical differences between stem(+) and stem(-) during flue-curing, all of which could be attributed to that, stems slowed down the rate of leaf dehydration.

## Data availability statement

The original contributions presented in the study are included in the article/Supplementary Material. Further inquiries can be directed to the corresponding authors.

## Author contributions

FS and BH conceived and designed the experiments; LM, WS, SC, FH, JC, BP participated in experiments and data analyses; LM and WS wrote the manuscript with inputs and guidance from BH and FS. All authors contributed to the article and approved the submitted version.

## Funding

This work was financially supported by the Agricultural Science and Technology Innovation Program (ASTIP-TRIC03).

## Conflict of interest

SC is employed by China Tobacco Shandong Industrial Co., Ltd.

The remaining authors declare that the research was conducted in the absence of any commercial or financial relationships that could be construed as a potential conflict of interest.

## Publisher’s note

All claims expressed in this article are solely those of the authors and do not necessarily represent those of their affiliated organizations, or those of the publisher, the editors and the reviewers. Any product that may be evaluated in this article, or claim that may be made by its manufacturer, is not guaranteed or endorsed by the publisher.
